# Paving the Way to Universal Health Coverage in Tanzania

**DOI:** 10.34172/ijhpm.9191

**Published:** 2026-06-10

**Authors:** Zaheer Rik Munshi, M. Connor Blue, Faraan O. Rahim, Erick Mahatara, Raghuram Reddy, Ketan Tamirisa, Arnav Ajay Jadav, Perisa Ashar, Anzibert Rugakingira

**Affiliations:** ^1^The College, University of Chicago, Chicago, IL, USA.; ^2^University of Pennsylvania Perelman School of Medicine, Philadelphia, PA, USA.; ^3^Harvard Medical School, Boston, MA, USA.; ^4^School of Medicine, KCMC University, Moshi, Tanzania.; ^5^Department of Humanities, Health and Society, Herbert Wertheim College of Medicine, Florida International University, Miami, FL, USA.; ^6^University of California, San Francisco, San Francisco, CA, USA.; ^7^Duke University, Durham, NC, USA.; ^8^The World Bank Tanzania, Dar es Salaam, Tanzania.

## Introduction

 Achieving universal health coverage (UHC)—high-quality, affordable healthcare for all without financial distress—is fundamental to equity, improved health, and financial protection. Since the 2010 World Health Organization’s (WHO’s) World Health Report, numerous countries, including Tanzania, have committed to achieving UHC.^1^ While notable progress has been made, particularly in areas such as maternal health and the reduction of under-five mortality, further efforts are required. Advancing UHC in Tanzania is crucial for fostering economic stability, reducing poverty, and building a healthier, more empowered population.

 Over the past six decades, Tanzania’s healthcare system has been shaped by key national and international milestones (See Table).^2^ These along with ongoing reforms have been crucial in supporting the well-being of the Tanzanian population and advancing progress toward UHC by 2030.^2^ Working towards UHC is vital for sustainable development, promoting economic stability, reducing poverty, and fostering a healthier, more productive population. In this opinion piece, we summarize Tanzania’s recent reforms and innovations in achieving UHC and discuss key health priorities and future directions (Figure).

**Table:** Understanding Milestones, Reforms & Innovations, and Initiatives

**Key Milestones**		**Positive Outcomes**
1967 Arusha Declaration		Emphasized self-reliance and equitable resource allocation
Alma-Ata Declaration of 1978		Established health as fundamental human right Advocated for primary healthcare
African Union’s Africa Health Strategy (2016–2030)		Committed governments to ensuring equitable healthcare access Aligned with the 2030 Agenda for Sustainable Development
2018 Astana Declaration		Reaffirmed global commitment to primary healthcare, particularly in resource-limited settings
**Key Health Reforms and Innovations**	**Functionality, Positive Outcomes**	**Ongoing Challenges, Mitigation Efforts**
**PHC Enhancements**		
DHFF	Improved health commodity availability, evidenced by increased funding for health facilities in a 2023 study Promotes accountability and local ownership by engaging communities in decision-making, tailoring services to local needs Improved structural quality, patient-perceived health system responsiveness, and maternal health service utilization Improved commodity financing and local decision-making autonomy Operationally, DHFF functions through a facility-level planning and accountability cycle in which facility management teams, in collaboration with governing committees and community representatives, develop annual plans and budgets and report expenditures through the FFARS^3,4^	Implementation challenges, including variable planning capacity, reporting workload, and ICT constraints Ongoing mitigation strategies include supportive supervision by Council Health Management Teams, routine performance monitoring, and continued training to strengthen financial management and accountability^3^ Governance and infrastructure challenges persist Necessitate stronger oversight and expanded healthcare worker training
**Medical Supply Chain and Technological Innovations**		
PVS	Enhance access to essential medicines by streamlining procurement through a single private supplier managing regional medical supplies under government oversight Pooled procurement approach consolidating orders at the district level, ensuring consistent, high-quality medicine supply, reducing stockouts, strengthening healthcare delivery Promoting sustainable healthcare financing	Challenges such as lack of uniformity in vendor performance across different regions in Tanzania, low fill rates to orders, late or unpredictable payments to vendors, and mixed stakeholder satisfaction due to implementation burden from increased administrative workload Ongoing mitigation strategies include PVS authorities and regional teams working to implement routine performance monitoring, strengthen vendor selection standards, and promote cascade training
GoTHOMIS	Streamline documentation, billing, and accountability Enhance resource allocation Reduce manual errors despite infrastructure and power challenges Other electronic medical record systems such as Jeeva, CTC2 database, and Afya care have also been utilized	The full impact of FFARS and GoTHOMIS is constrained by persistent ICT and human resource gaps, particularly at rural primary health facilities and district hospitals where digital infrastructure is weakest^6^ Key bottlenecks include low and unstable internet bandwidth, limited hardware availability such as insufficient computers per facility, intermittent electricity supply, and limited technical support capacity, which collectively affect system usability and real-time data reporting^4^ In practice, these constraints most acute in remote and underserved regions where ICT infrastructure, power reliability, and managerial support for digital systems remain uneven across districts^9^ To address staffing shortages and digital literacy gaps, the Ministry of Health and implementing partners have introduced cascade training, on-the-job mentorship, and supportive supervision through Council Health Management Teams to strengthen system adoption and financial reporting competencies under DHFF and FFARS implementation^4,9^ Emerging evaluations suggest that GoTHOMIS and FFARS have improved documentation accuracy, financial accountability, and facility-level resource tracking, although they have also increased reporting workload and usability demands for frontline health workers and facility managers, underscoring the importance of incorporating user feedback into ongoing system refinement^4^ Current scale-up strategies emphasize phased national rollout, integration with DHFF workflows, continued capacity building, and government-led stewardship to ensure long-term sustainability and interoperability within Tanzania’s broader digital health architecture^4,9^
FFARS	Enhances financial oversight by enabling real-time tracking of revenue and expenditure across >5000 primary health facilities, improving resource allocation^4^ Uses standardized budget codes and accounting classifications consistent across facility and council levels and compliant with IPSAS financial reporting standards Improved visibility and tracking of health commodity funding and expenditures at primary health facilities	
**Key Ongoing Healthcare Initiatives**		
M-Mama	President Samia Suluhu Hassan launched M-Mama (2022) A maternal health initiative in Tanzania to reduce maternal and neonatal mortality	Improving emergency care access Utilizing digital technology for efficient transport and remote triage Significantly enhancing health outcomes for pregnant women and newborns^13^
Maternal and Perinatal Death Review Initiative	National surveillance and systematic review of maternal and neonatal deaths^13^	This national initiative contributed to a 35% mortality reduction^13^
Digital Provider Communication (eg, WhatsApp clinical coordination)	Real-time specialist consultation and provider communication in severe cases	Improved reproductive health outcomes, with only 4.6% of serious cases resulting in death after requesting technical support^13^ This decline is also attributed to increased antenatal care attendance, fewer teenage pregnancies, more facility-based deliveries, and a higher proportion of births attended by skilled professionals

Abbreviations: PHC, primary healthcare; DHFF, Direct Health Facility Financing; FFARS, Facility Financial Accounting and Reporting System; ICT, information and communication technology; PVS, Prime Vendor System; GoTHOMIS, Government of Tanzania Hospital Management Information System; IPSAS, International Public Sector Accounting Standards.

**Figure:**
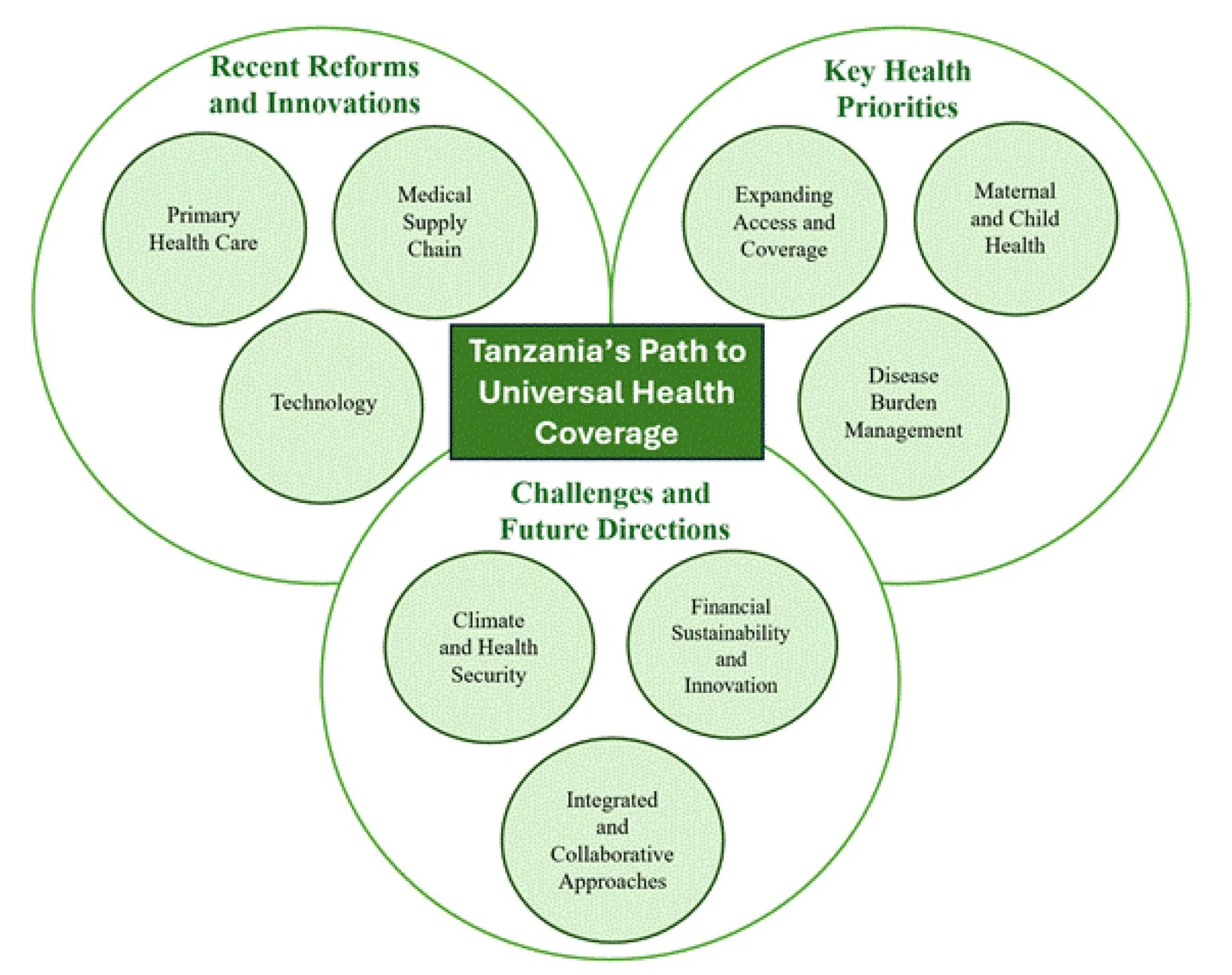
Conceptual Framework for Tanzania’s Path to Universal Health Coverage.

## Recent Reforms and Innovations

###  Primary Healthcare Enhancements

 Since 2015, the Late President of Tanzania, John Pombe Magufuli led major upgrades in Tanzania’s primary healthcare infrastructure, strengthening surgical and diagnostic capacity. From 2021, under the President Samia Suluhu Hassan, increased funding further expanded facilities, equipment, bed capacity, and emergency services. However, achieving UHC still requires more functional facilities and trained surgical personnel. In 2017, the government implemented Direct Health Facility Financing (DHFF), a decentralization initiative providing funds directly to health facilities to enhance resource management and service delivery.^3^ See Table for functionality and improvements.

 Despite significant progress, the financial and human resource foundations of Tanzania’s health system remain under pressure. As of 2022, the total health expenditure reached approximately TSh 6.83 trillion (US $2.9 billion), representing 5% of the national gross domestic product, a notable increase from 4% in 2020.^5^ However, health sector allocation as a share of the total government budget stood at 11.2% in fiscal year 2021/2022, still falling short of the 15% Abuja Declaration target.^6^ These budgetary constraints are compounded by a severe shortage of human resources for health. In 2023, only 36% of the required 348 923 health workers were available, resulting in a national workforce deficit of 64%.^7^ The current human resources for health density of roughly 8.42 per 10 000 population remains significantly below the WHO benchmark of 22.8 required for effective UHC delivery.^8^

###  Medical Supply Chain and Technological Innovations

 Other recent advancements in Tanzania have included innovations in medical supply chains and technology. Public-private partnerships like the Jazia Prime Vendor System (PVS) enhance access to essential medicines (See Table for benefits and challenges).

 Moreover, to improve healthcare efficiency and achieve UHC goals, Tanzania implemented two digital systems: the Government of Tanzania Hospital Management Information System (GoTHOMIS) in 2015 and the Facility Financial Accounting and Reporting System (FFARS) alongside the DHFF initiative in 2017–2018 (See Table for details).^4^

## Addressing Key Health Priorities

###  Expanding Access and Coverage for Underserved Populations

 Tanzania’s UHC Service Coverage Index improved from 20 in 2000 to 43 in 2021 due to increased service availability, financial protection, and access to essential healthcare.^10^ Expanding health insurance coverage through initiatives such as the National Health Insurance Fund and Community Health Fund has enabled at least 32% of the population to be covered by 2018.^11^ Although the percentage is comparative to countries like Kenya and Ethiopia, it is significantly lower to that of Rwanda, Tunisia, and Seychelles, countries that have been able to achieve more than 90% coverage.^12^ However, reaching full UHC requires closing coverage gaps among underserved populations who may be unable to contribute to insurance schemes. This calls for continued investment, policy reforms, and equitable financing to ensure no one is left behind.

###  Maternal and Child Health

 Tanzania achieved an impressive 80% reduction in maternal mortality between 2016 and 2022 through several key interventions.^13^ The reduction percentage is significantly higher than the overall sub-Saharan region’s average (56%). The majority of the region was able to reduce maternal mortality by improving maternity care, while several like Kenya, Botswana, and Nigeria emphasized fertility reduction; Tanzania uniquely combined both.^14^ To illustrate, interventions included nearly quintupling Emergency Obstetric and Newborn Care centers, exceeding WHO recommendation of one Comprehensive Emergency Obstetric and Newborn Care facility per 500 000 people, and implementing mentorship programs where senior gynecologists trained junior staff in remote areas.^13^ Sustaining these gains (See Table for more initiatives) requires continued investment in healthcare and critical infrastructure, including rural road networks, energy access, and water supply.

###  Disease Burden Management

 Tanzania is experiencing a rising burden of non-communicable diseases (NCDs), now responsible for about one-third of all deaths and projected to surpass communicable disease mortality.^15^ The government has responded by establishing the National NCD program, developed strategic plans and research frameworks, and strengthening partnerships with academic institutions and non-governmental organizations (NGOs). At the same time, recurrent infectious threats, including the 2023 and 2025 Marburg virus outbreaks, underscore the need to reinforce infection prevention and control measures and disease surveillance.^15^ Addressing this dual burden requires equitable resource allocation and an integrated healthcare system that combines prevention, emergency preparedness, and community engagement. Although Tanzania (34%) reports a lower proportion of NCD-related death than Mauritius (88.4%), Rwanda (50.4%), and South Africa (51.3%), the burden remains substantial and comparable to Zambia (34.8%) and Uganda (35.6%).^16^ Note, although Tanzania falls somewhat in the moderate region for NCDs, however it faces significantly high burden management for communicable diseases like Malaria and neglected tropical diseases.^17^

 Tanzania is integrating NCD services into primary healthcare platforms through routine screening for hypertension, diabetes, and key risk factors during outpatient and maternal health visits. Implementation follows WHO PEN-aligned protocols and task-sharing to nurses and clinical officers.^18^ Priority interventions include standardized treatment pathways, risk-factor counseling, cervical cancer screening, and improved availability of essential diagnostics and medicines. Progress should be tracked through screening coverage, treatment initiation, control rates, and stockout frequency, alongside communicable disease metrics. Continued investment in workforce training and referral systems remains essential to strengthen PHC capacity for both diseases and burdens.

## Challenges and Future Directions

###  Climate and Health Security

 Addressing climate change’s health risks requires climate-resilient health systems and pandemic preparedness, as emphasized by WHO and the International Health Regulations. A climate-resilient health system is one that can anticipate, respond to, cope with, recover from, and adapt to climate-related shocks while sustaining essential health services. In Tanzania, this framework aligns six core building blocks: (1) governance and leadership through integration of climate resilience into national health strategies and cross-sectoral coordination; (2) workforce development with climate-focused training and surge capacity; (3) service delivery resilience through climate-proofed infrastructure (electricity, water, cooling systems); (4) health information systems with climate-health data integration, early warning systems, and real-time surveillance for climate-sensitive diseases; (5) access to essential medicines and technologies with climate-adapted supply chains; and (6) sustainable financing for climate adaptation interventions.^19^

 Tanzania’s Health National Adaptation Plan integrates climate resilience into health policies, prioritizing disease surveillance, emergency response, and climate-informed strategies to address vulnerabilities like malaria and cholera.^9^ Specific interventions being pursued include vulnerability and capacity assessments of the health systems, establishment of contingency plans for essential services during extreme weather events, intensified surveillance systems targeting climate-sensitive diseases such as malaria and cholera, infrastructure upgrades to withstand flooding and heat stress, and means of unified surveillance platforms aimed at monitoring both climate hazards and disease outbreaks, coordinated emergency preparedness protocols that address climate-related health emergencies alongside pandemic threats, and cross-sectoral coordination mechanisms linking health, environment, agriculture, and disaster management sectors.^19^ Aligning with WHO strategies for pandemic preparedness, Tanzania secured a $25 million World Bank Pandemic Fund grant with US support, which helped contain a 2023 Marburg virus outbreak in under three months.^20^ Tanzania’s August 2023 Joint External Evaluation of International Health Regulations core capacities showed significant progress across all 19 technical areas, with strengths in political commitment and One Health platforms.^21^ However, areas for improvement include strengthening the National Action Plan for Health Security, legal frameworks, leadership, resource allocation, and capacity building to ensure long-term resilience.

###  Financial Sustainability and Innovation

 The inadequacy of traditional healthcare funding mechanisms is particularly evident in emerging economies striving to meet their infrastructure demands. Tanzania faces such challenges, where a dependence on donor funding and fragmented insurance schemes impede progress towards UHC. The existing the National Health Insurance Fund, Community Health Fund, and private micro-insurance programs suffer from low enrollment and inadequate risk pooling.^22^ This suggests that social health insurance alone may not overcome the financial and structural barriers to UHC. Blended financing models, which strategically combine private equity, government funding, and philanthropic capital, offer a potential pathway to optimize resource allocation. Overall, expanding insurance coverage and integrating risk-pooling mechanisms are key to achieving financial sustainability and broader healthcare access.

 Aligning with the international health financing framework is essential to integrate service and to ensure sustainability. Sustainable financing requires pre-payments and risk pooling to prevent catastrophic health expenditures and impoverishment, while ensuring equitable resource distribution (WHA58.33).^23^ Systems should minimize out-of-pocket payments and support transition to UHC, maintaining service quality and balanced investment in health promotion, prevention, rehabilitation and care provision (WHA64.9).^24^ Tanzania’s shift from donor dependence to domestic resource mobilization reflects these principles through the Universal Health Insurance Act (2023), which mandates insurance enrollment and strengthens prepayment and risk pooling across formal and informal sectors.^25^ The DHFF model enhances sustainability by enabling facilities to manage revenues directly, reducing reliance on vertical donors and reinforcing government stewardship of health financing.^25^

 However, the feasibility and scalability of these reforms remain uneven, particularly in rural and underserved regions where infrastructure, workforce capacity, and digital connectivity are weakest.^26^ While initiatives such as DHFF, GoTHOMIS, and expanded PHC services show promise, their implementation depends on reliable electricity, trained personnel, supply chain stability, and sustained financing at the district level, all of which are recognized core health system building blocks for effective service delivery. In many remote settings, shortages of skilled health workers, limited information and communication technology (ICT) infrastructure, and transportation barriers may constrain the scale-up and long-term sustainability of these reforms, particularly in geographically remote districts with uneven health workforce distribution and infrastructure capacity.^26^ Therefore, phased implementation, targeted rural investment, and context-specific capacity building will be essential to ensure equitable and scalable UHC progress across regions.

###  Collaborative and Integrated Approaches

 Leveraging the One Health approach, Tanzania has established a well-structured and functional multi-sectoral coordinating mechanism with strong capabilities in preventing, detecting, and responding to health threats.^27^ More specifically, by utilizing existing tools like animal vaccinations and combining expertise from human, wildlife, and environmental health sectors, this strategy has allowed Tanzania to tackle both emerging and re-emerging zoonotic diseases.^27^ Furthermore, Tanzania’s healthcare system is strengthened by strategic regional and global partnerships that provide vital financial support, capacity-building initiatives, and infrastructure development. Notably, the World Bank has committed over $750 million through the Tanzania Inclusive and Resilient Growth Development Policy Financing and the Tanzania Maternal and Child Health Investment Program to bolster healthcare resilience and enhance maternal and child health services.^28^ Other key partners offering significant financial and technical assistance include the United States Agency for International Development, the Global Fund, the WHO, and the Gates Foundation. Other developing countries such as Ghana with the National Health Insurance Scheme, Rwanda, Botswana, Algeria, Tunisia, and others have made significant strides to achieving UHC.

 Beyond government action, the success of Tanzania’s health and climate initiatives relies heavily on a robust network of civil society organizations and NGOs. These stakeholders play a dual role in service delivery and policy advocacy. For instance, in 2023, over 2720 civil society organizations were documented as actively contributing to the implementation of Sustainable Development Goals (SDGs), particularly in health (SDG 3) and climate action (SDG 13).^29^ Organizations such as Tanzania NCDs Alliance advocate for NCDs inclusion in UHC, while others like EngenderHealth and International Center for AIDS Care and Treatment Programs have historically provided the technical bridge between national policy and community-level outreach. These partnerships are formalized through the Sector-Wide Approach, which ensures that NGO activities align with national priorities and fill critical gaps in rural services provision.^30^

###  Transitioning to Domestic Sustainability

 The recent withdrawal and restructuring of US foreign aid (specifically the United States Agency for International Development and the United States President’s Emergency Plan For AIDS Relief) in early 2025 has created a critical gap, particularly in the HIV/AIDS and maternal health sectors, where US funding historically accounted for nearly 70% of disbursed health aid.^31^ This transition has led to the cessation of key outreach programs and significant loss of healthcare positions previously supported by donor grants. To strengthen policies with minimal external reliance, Tanzania is prioritizing the Health Sector Strategic Plan V (2021-2026), which emphasizes domestic resource mobilization through the Universal Health Insurance Act of 2023. By mandating health insurance and scaling the DHFF model, the government aims to empower local facilities to manage their own revenue and reduce dependence on vertical donor funding.^30^

 Beyond financing and donor transition, recent developments also underscore the importance of integrating palliative care into primary healthcare (PHC), strengthening digital health governance, and addressing the ethical burden on healthcare workers in the pursuit of UHC in Tanzania. As the burden of NCDs rises, PHC-level palliative care is increasingly recognized as essential for person-centered and continuous care delivery and is explicitly endorsed by WHO as a core component of UHC-oriented health systems.^32^ In parallel, the expansion of digital health systems, including electronic medical records and national digital platforms, reflects a broader shift toward data-driven health system strengthening; however, equitable digital infrastructure, ethical data governance, and sustained workforce training remain critical to avoid widening urban–rural disparities. Persistent workforce shortages and high service demands further raise concerns regarding burnout, moral distress, and ethical prioritization in resource-constrained settings, which may affect the long-term sustainability of UHC implementation.

## Conclusion

 Tanzania has advanced towards UHC through strategic investments and key reforms like DHFF, GoTHOMIS, and Jazia PVS, enhancing efficiency, accountability, and access. Focus on maternal health, child well-being, disease management, and digital integration underscores its commitment to equitable care. However, expanding insurance, building climate-resilient infrastructure, and tackling NCDs demand sustained attention. Achieving financial sustainability, stronger governance, and cross-sector collaboration are crucial to close coverage gaps. To maintain momentum towards 2030, Tanzania must prioritize prevention, equity, and innovation, requiring continued commitment from policy-makers, providers, and partners to build an inclusive and resilient healthcare system and ensure quality care for all.

## Disclosure of artificial intelligence (AI) use

 This document was created with assistance from Google Scholar for literature review.

## Ethical issues

 Not applicable.

## Conflicts of interest

 Authors declare that they have no conflicts of interest.
